# Spatial variability of Spanish sardine (*Sardinella aurita*) abundance as related to the upwelling cycle off the southeastern Caribbean Sea

**DOI:** 10.1371/journal.pone.0179984

**Published:** 2017-06-27

**Authors:** Digna Rueda-Roa, Jeremy Mendoza, Frank Muller-Karger, Juan José Cárdenas, Alina Achury, Yrene Astor

**Affiliations:** 1 College of Marine Science, University of South Florida, Saint Petersburg, Florida, United States of America; 2 Instituto Oceanográfico de Venezuela, Universidad de Oriente, Cumaná, Estado Sucre, Venezuela; 3 The Nature Conservancy, Caracas, Distrito Capital, Venezuela; 4 Estación de Investigaciones Marinas, Fundación La Salle de Ciencias Naturales, Punta de Piedras, Estado Nueva Esparta, Venezuela; Aristotle University of Thessaloniki, GREECE

## Abstract

The *Sardinella aurita* fishery off northeastern Venezuela, region of seasonal wind-driven coastal-upwelling, accounts for 90% of the Caribbean Sea small pelagic catch. This law-protected artisanal fishery takes place up to ~10 km offshore. The spatial distribution, number of schools, and biomass of *S*. *aurita* were studied using eight hydro-acoustic surveys (1995–1998). The study included the analysis of satellite-derived sea surface temperature and chlorophyll-a. Surveys were grouped by strong, weak, and transitional upwelling seasons. Relationships between these observations were analyzed using Generalized Additive Models. Results show that during the primary upwelling season (January-May) sardines were widely distributed in upwelling plumes that extended up to 70 km offshore. In the other hand, during the weak upwelling season (September-October) higher sardine densities were found within 10 Km off the coastal upwelling foci. The number of small pelagic schools was directly correlated with small pelagic densities; however, regardless of the season, higher numbers of small pelagic schools were always closer to the shoreline, especially during warm conditions. These two behaviors increase the availability and catchability of sardines for the artisanal fishery during the warm season, regardless of the total stock size. Using this evidence, we pose the hypothesis that the collapse of the regional *S*. *aurita* fishery in 2005 was due to a combination of stressful habitat conditions sustained since 2004. These included bottom-up factors due to food scarcity caused by weak upwelling, combined with top-down stress due to overfishing, as sardines accumulated in narrow diminished upwelling plumes located close to the coast. The increased catchability within easily accessible upwelling foci led to the demise of this biological resource, which as of 2014 had not yet recovered. Environmental conditions affecting the sardine habitat needs to be taken into account for the management of this stock. For example, during years with weak upwelling, special measures should be taken during the warm season on the second half of the year to avoid further pressure on the stock.

## 1. Introduction

**Fig 1:**
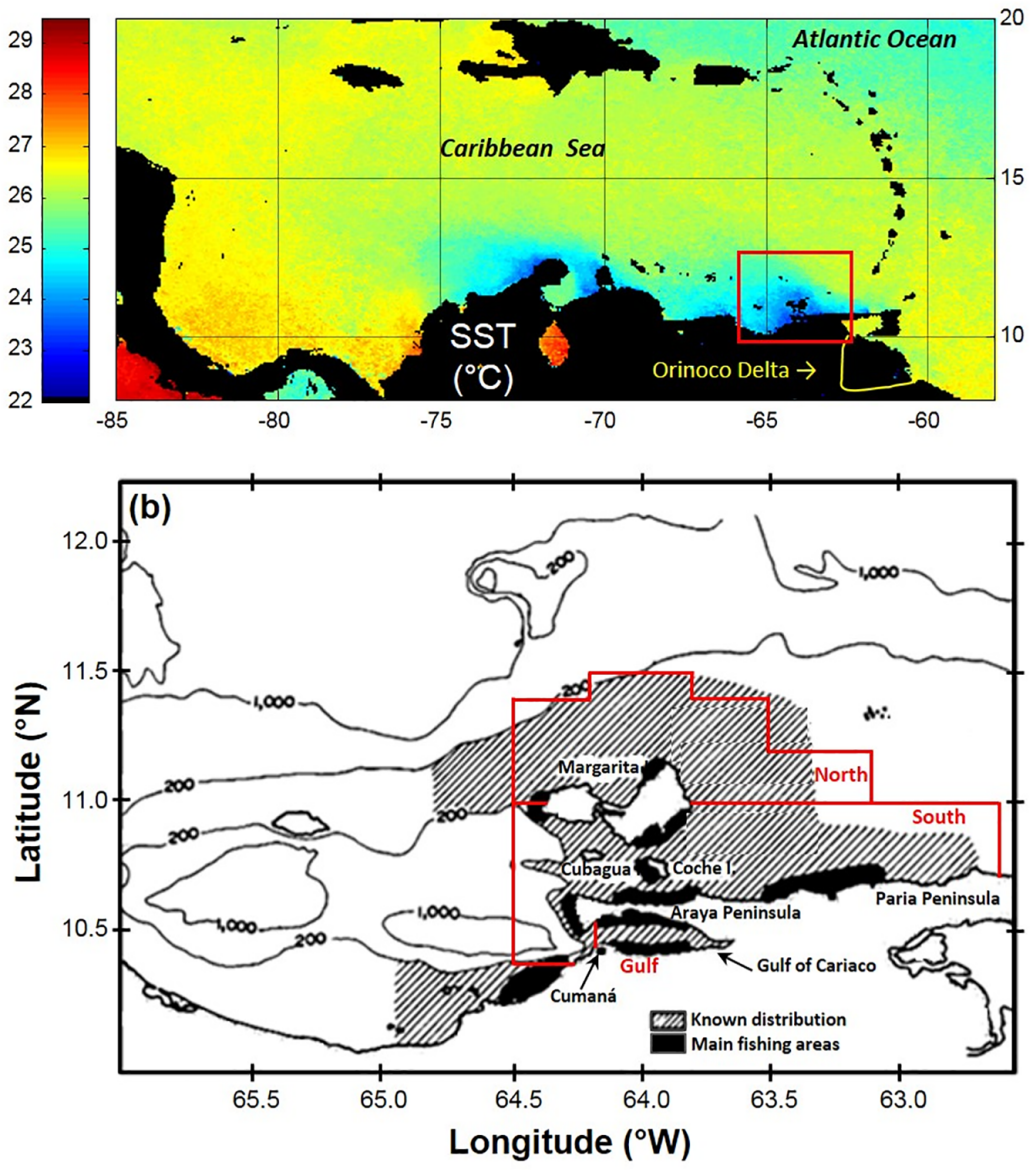
Southern Caribbean Sea upwelling and region of interest in the southeastern Caribbean Sea. (a) Caribbean Sea satellite Sea Surface Temperature (SST) climatology for February (1994–2009) showing the peak of the seasonal upwelling in the southern Caribbean; the region of interest off northeastern Venezuela is marked with a red rectangle. (b) Known distribution of *Sardinella aurita* (hashed lines) and main fishing areas (black) off northeastern Venezuela. Ground-truthing of the acoustic backscattering echo sound surveys was done for every VECEP survey by exploratory fishing within the three areas delimited in red: 1) Gulf of Cariaco, 2) South Area, and 3) North Area.

The southern Caribbean Sea (Fig 1A) is an area of pronounced seasonal coastal upwelling [1, 2]. Within this upwelling system, the continental shelf off northeastern Venezuela (Fig 1B) is one of the most important fishing grounds in the Caribbean Sea [3, 4]. This relatively small shelf (Fig 1B) carries a Spanish sardine (*Sardinella aurita*) biomass of 0.6–1.3 million tons [5, 6, 7]. The Spanish sardine fishery in this area represents almost 90% of the small pelagic fish catch for the Caribbean Sea [3, 4]. Despite the broad geographic distribution of sardine over this continental shelf, fishing takes place primarily within ~10 km of the coast as it is carried out from small artisanal boats. The artisanal nature of this fishery is protected by law [8, 9, 10].

Changes in habitat conditions have profound impacts on fish spatial distribution [11]. Our research sought to evaluate how the spatial distribution of *S*. *aurita* responds to changes in their habitat due to the seasonal upwelling in the southeastern Caribbean Sea. Historical acoustic surveys [5, 12–17] had provided estimates of sardine abundance and distribution in the region at different times. For this work, we used a series of eight acoustic surveys performed by the “Technical Cooperation Program for fisheries between the European Union and Venezuela, Colombia, Ecuador and Perú” (UE-VECEP ALA 92/43, [18]). The VECEP acoustic surveys off northeast Venezuela were carried out between 1995 through 1998, coinciding with the availability of satellite-derived Sea Surface Temperature (SST) imagery. Satellite SST serves as a proxy for the upwelling intensity and geographic extent in this area [19].

In a previous analysis of the VECEP surveys, Cárdenas and Achury [6] found no evident correlations between Spanish sardine biomass and *in situ* profiles of water temperature and chlorophyll-a concentration obtained during the VECEP surveys. Those analyses were considered challenging due to the aggregative behavior of sardines [6]. Aggregation behavior of sardines off South Africa causes a patchy spatial distribution, with a significant portion (90%) of sardine biomass located in a small fraction (<10%) of the acoustic transects [20, 21]. The contagious distribution of clupeids in schools and aggregations makes it a challenge to analyze relationships between their abundance and sparse environmental observations. We took advantage of the synoptic coverage of satellite SST and by using a different approach in the data analysis using histograms and Generalized Additive Models (GAM) to analyze the spatial distribution of sardines related to the upwelling cycle. These results provided insights on the causes of the crash of the sardine fishery off northeast Venezuela in 2005 [3, 22].

## 2. Study area, data and methods

### 2.1 Study area

The study area is located in the southeastern Caribbean Sea, off northeastern Venezuela (10–12°N and 61.5–65°W; Fig 1). The continental shelf in this region is roughly oriented in an east-west direction. The offshore extension of the shelf ranges from 5 to 100 km, with depths of between 7–115 m before dropping more steeply into the Caribbean Basin [9] (Fig 1B). The prevailing Trade Winds and the coastline orientation and shelf topography favor year-round wind-induced coastal upwelling, with seasonal intensification between December and April, during the time of stronger Trade Winds [1, 2, 23]. A shorter and less intense secondary upwelling event occurs around mid-July [19].

**Fig 2:**
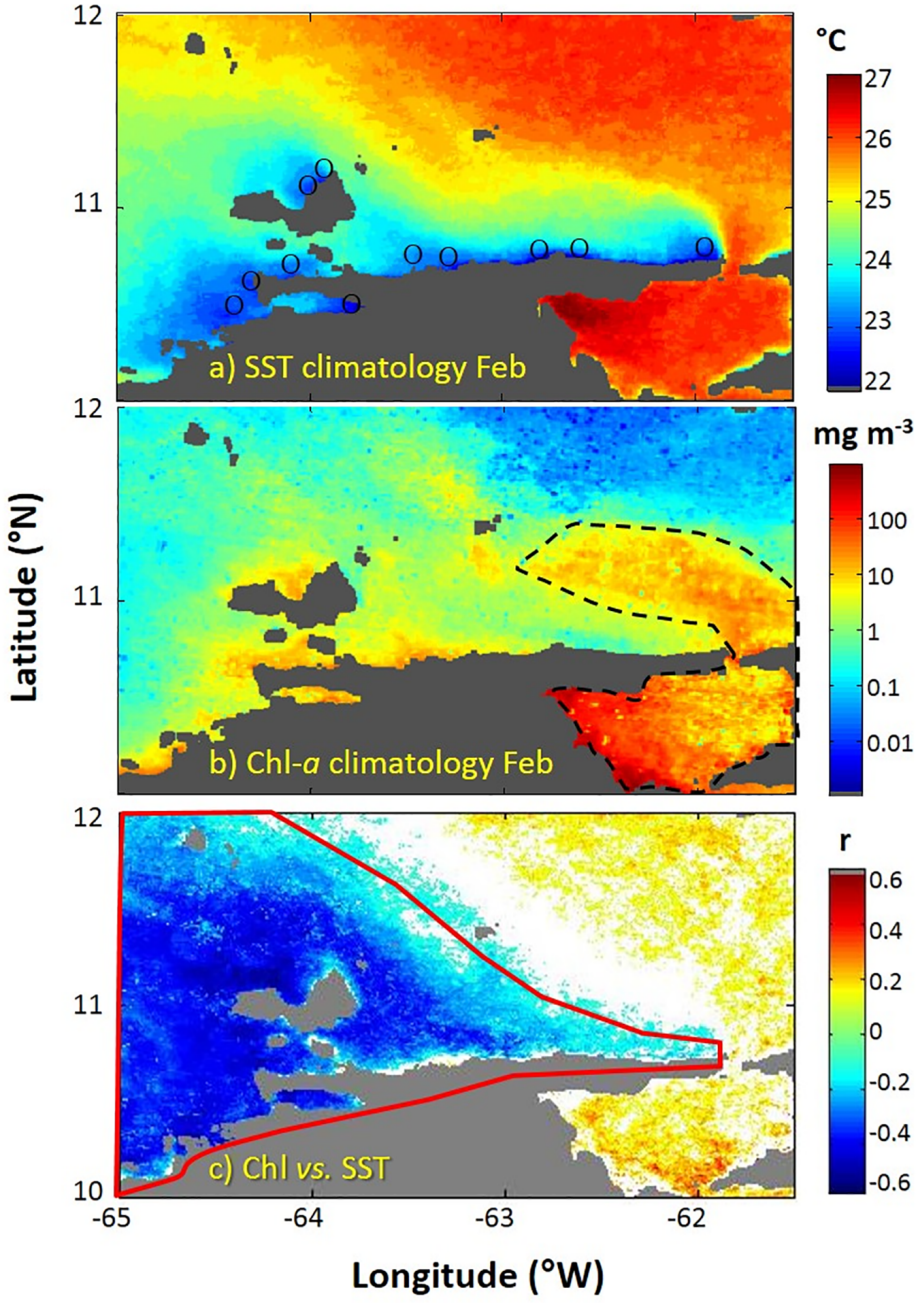
Sea surface temperature and chlorophyll-a conditions in the study area. The study area (10°N to 12°N and 61.5°W to 65°W) is a region affected by strong upwelling during the first half of the year, and also by the Orinoco River discharge plume to the east. (a) SST climatology for February (1994–2009) shows strong upwelling conditions, including the upwelling plumes (blue and green colors), the upwelling foci (circles, from Rueda-Roa and Muller-Karger [19]), and warmer SSTs to the north in the open Caribbean Sea and to the east in the region of the Orinoco river plume. (b) Chlorophyll concentration (Chl) climatology for February (1998–2009) showing high Chl within the upwelled waters. The eastern region shows false higher Chl due to Colored Dissolved Organic Matter (CDOM) in the Orinoco River plume (delimited by the broken black line). (c) Correlation coefficient between weekly time series of SST and logChl (1998–2009). Spatial averages for this study were computed only for the area to the west delimited with the red polygon to avoid inclusion of the Orinoco plume, i.e. the area with positive SST-Chl correlations or not significant (p>0.05) negative correlations.

The warmest season (September-November), show sea surface temperatures ~1°C cooler than the central Caribbean Sea due to a weak coastal upwelling process [2, 19]. The coastal upwelling region shows relatively high levels of primary production (0.2 to 3 gC m^2^ d^-1^; [24]). The discharge from the Orinoco River, which enters the Caribbean Sea to the east of the study area, adds complexity to the hydrological and bio-optical conditions of the sardine habitat (Fig 2). Maximum river discharge occurs between July-October and the river plume reaches as far north into the Caribbean Sea as Puerto Rico [25, 26].

### 2.2 Acoustic survey data

**Fig 3:**
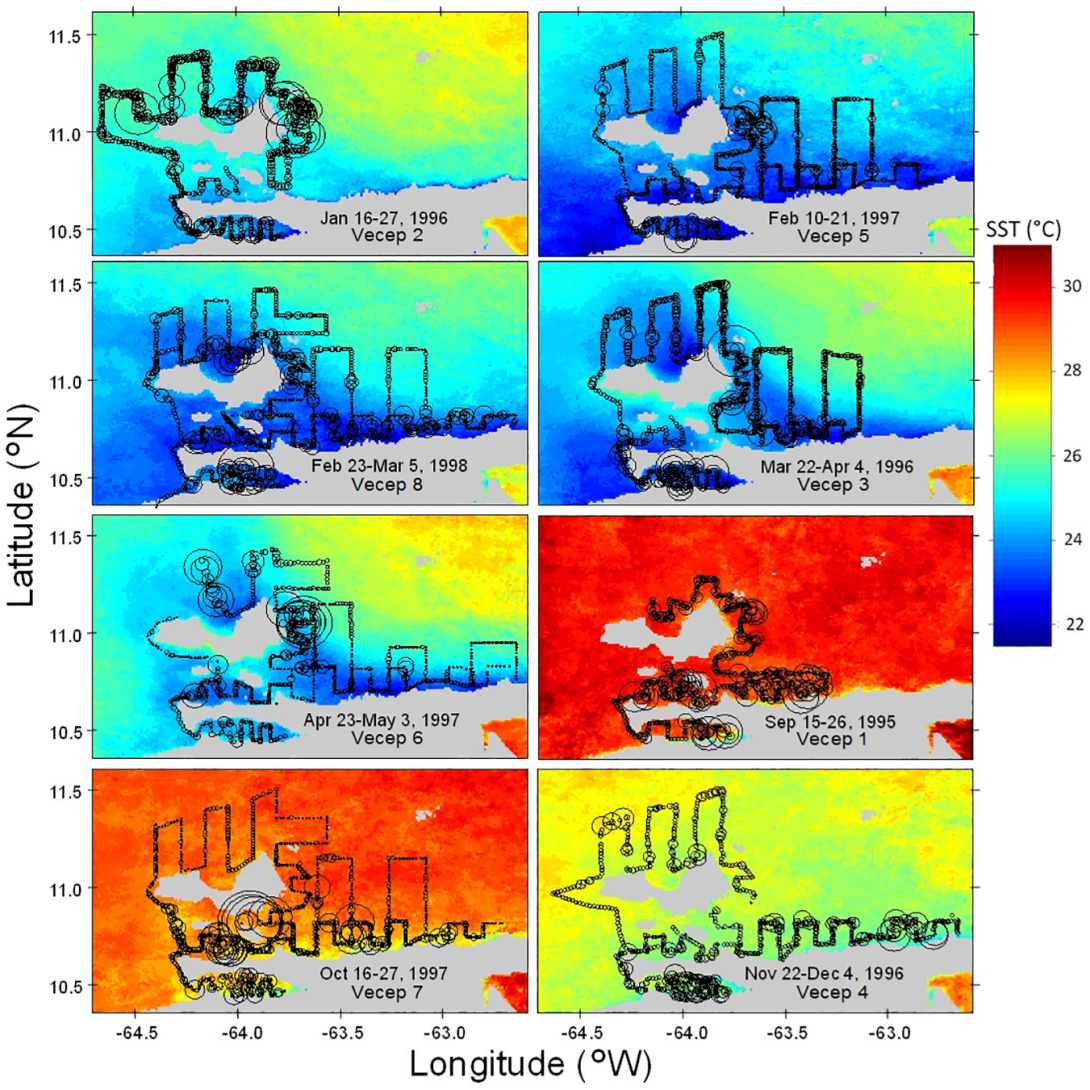
Spanish sardine relative abundance index (sardine-NASC) and SST during each VECEP survey period. Sardine-NASC is proportional to circle size along the tracks of each VECEP acoustic backscattering echo sound survey. SST was averaged within the days of the survey indicated in each panel. The order of the panels is according to the month when the survey was conducted.

Spanish sardine density and spatial distribution were obtained from eight acoustic surveys performed during 1995–1998 with the R/V Hermano Ginés (Fundación La Salle de Ciencias Naturales, Venezuela), under the Regional Technical Cooperation Fishing Programme VECEP (Venezuela, Colombia, Ecuador, Peru) UE-VECEP ALA 92/43 between the European Union and Venezuela [18]. We refer to the acoustic fish surveys used in this study simply as the VECEP surveys. Dates for each survey are shown in Fig 3.

Pelagic fish acoustic backscattering strength was measured with a split-beam echo sounder SIMRAD EK500 at 38 and 120 kHz. The study area is characterized by high plankton concentration and relatively shallow depths around the study area (from 10 to 150 m). Most of the processing was based on the data acquired at 120 kHz, since 120 kHz allows a better discrimination of fish echoes from plankton´s and it has a range of about 100 m, in deeper areas the 120 kHz system was mainly used as an aid in the discrimination of fish traces recorded by the 38 kHz system [27, 28]. The navigation velocity for the echo-surveys was 7 knots (12.9 km h^-1^) and the elementary sampling distance unit (ESDU) was one nautical mile (1852 m). The tracks of each cruise were measured twice during day and night conditions. Calibrations of the echosounder were performed before every survey following the sphere protocol described in Foote et al. [29].

Acoustic fish assessment uses the echo-integration method, which estimates the acoustic density of the target fish along the water column and within a given area, being the acoustic density proportional to the fish density. The result of this echo-integration, is the nautical-area scattering coefficient, or NASC (also called s_A_, with units in m^2^ nmi^-2^) [30]. To minimize contamination from other signals (i.e. demersal fish going up during night time) the echo-integration was done up to 2 m above the bottom, and values exceptionally high and not explained by the presence of pelagic fish were eliminated. This resulted in a value for small pelagic NASC (pelagic-NASC) for every nautical mile along the survey track. The number of small pelagic schools was also integrated every nautical mile. Exploratory fishing using semi-pelagic trawls were performed during each survey to ground-truth the acoustic backscattering echo-sound surveys. *Sardinella aurita* cannot be distinguished from other small pelagic by visual inspection of the echogram; therefore, its density estimation (sardine-NASC) was calculated by its proportion in the fish community, obtained as the average of the trawling fishing results within three delimited areas (Fig 1B): (1) The Gulf of Cariaco, (2) The South Area (10.6°N to 11°N, and (3) The North Area (11° N to 11.4° N off northern Margarita Island). The Gulf of Cariaco features an upwelling focus in its southeastern extreme (Fig 2A) and it is considered an important nursery for Spanish sardine [31]. The South Area also shows important upwelling foci along the northern coasts of the Paria and Araya peninsulas (Fig 2A), with upwelling plumes extending NW. The eastern of the South Area is affected by the Orinoco River discharge on a seasonal basis (Fig 2). Open waters of the North Area receive the input of the upwelling plumes from the South Area and discharge from the Orinoco River (Fig 2). When exploratory fishing was not possible in an acoustic survey area, the factors used were the average obtained for that area from other VECEP surveys. The Spanish sardine factors varied from 0.1 to 1 with an average of 0.54 [6].

### 2.3 Satellite sea surface temperature and chlorophyll-a concentration

Satellite-derived Sea Surface Temperature (SST) and Chlorophyll-a concentration (Chl) were extracted for the study area (Fig 2). High resolution SST imagery (~1 km^2^ pixel) from the Advanced Very High Resolution Radiometer (AVHRR, National Oceanic and Atmospheric Administration), were collected using a ground-based antenna located at the University of South Florida (St. Petersburg, Florida, U.S.A.). SST was derived using the Multi-Channel Sea Surface Temperature (MCSST) split-window techniques [32, 33, 34]. The nominal accuracy of AVHRR SST retrievals is in the range of ±0.3 to ±1.0°C [35, 36]. AVHRR imagery contains false cold pixels due to cloud contamination; therefore, to improve data quality we implemented a cloud filter similar to that of Hu et al. [37]. The filtered daily images were used to calculate weekly averages and a long-term weekly SST climatology (1994–2009, which we refer to here as “climatologies”), as well as averaged SST for the duration of each VECEP cruise (see surveys dates in Fig 3).

To assess whether Spanish sardine has a tendency to aggregate close to upwelling or thermal fronts, we calculated SST gradients (SST-gradient) from the SST imagery covering each acoustic survey. SST-gradient was computed at each grid point (i, j) by subtracting the SST from the pixels to the north-south and east-west of the grid point using the following equation:

$$SSTgradient\left(i,j\right)=\sqrt{{\left((SST\left(i+1,j\right)-SST\left(i-1,j\right))/2km\right)}^{2}+{\left((SST\left(i,j+1\right)-SST\left(i,j-1\right))/2km\right)}^{2}}$$ (1)

We used high resolution satellite Chlorophyll-a concentration (Chl, 1 km^2^ pixel), produced with the default NASA OC4 chlorophyll algorithm [38] from images collected with the Sea-viewing Wide Field-of-view Sensor (SeaWiFS). Images were downloaded from the NASA Goddard Space Flight Center Distributed Active Archive (September 1997 to December 2009) and mapped to a uniform space grid scale using Matlab routines. Weekly Chl time series composites and weekly long-term means (1998–2009) were computed as geometric means, i.e. based on the mean of log-transformed chlorophyll-a data (logChl). An inverse log (anti-log) was applied to obtain final chlorophyll concentration values. SeaWiFS Chl was concurrent only for two acoustic surveys, namely VECEP 7 and 8, which were carried out during a weak and a strong upwelling seasons, respectively.

We used correlations between weekly time series of SST and Chl (1998–2009, Fig 2C) to delimit the area influenced by upwelling from the area influenced by the Orinoco discharge (more details in Rueda-Roa and Muller-Karger [19]). The area dominated by the Orinoco River discharge is characterized by warm SST (Fig 2A) and also by false high values of satellite Chl (Fig 2B) due to the presence of elevated concentrations of riverine Colored Dissolved Organic Matter (CDOM) [39, 40]. The boundary between the upwelling and the river plumes was determined by a linear correlation between weekly time series of SST and logChl (Fig 2C). The correlation coefficients showed a negative/positive relationship in the areas influenced by the upwelling/Orinoco plume (Fig 2C). To calculate the spatial averages of SST and Chl related solely to upwelling dynamics we masked out the area affected by the Orinoco plume, which was defined as the region with positive correlations or no significant negative correlation (p>0.05) between SST and Chl (Fig 2C) as per Rueda-Roa and Muller-Karger [19]. For each VECEP cruise and for the weekly SST and Chl climatology, spatial averages and standard deviations were calculated for the area defined in Fig 2C, excluding the Orinoco plume.

The annual catch of Spanish sardine was obtained from the Global Capture Production Dataset from the Food and Agriculture Organization, FAO [4]. Annual averages of SST and CHL, spatially-averaged in the polygon area of Fig 2C, were contrasted with annual catches of *Sardinella aurita* in eastern Venezuela.

### 2.4 Data analysis

We used the following variables obtained for each sample collected during the VECEP surveys: survey number, sampling time, latitude, longitude, sardine Nautical area scattering coefficient (sardine-NASC), number of small pelagic schools (Schools) and sampling area number 1, 2, or 3 (Area, Fig 1B). At each nautical mile along the acoustic tracks we derived several products from the satellite images, namely: SST, SST gradient, distance to the nearest upwelling focus (upw-foci, Fig 2A), and Chl (again, available only for VECEP 7 and 8).

To overcome the challenge of the aggregation behavior of sardines and visualize the distribution of sardine-NASC related to the seasonal intensity and dispersion of upwelling (SST), we first computed histograms of SST from satellite data extracted along the cruise track. Then geometric average of sardine-NASC and average of Schools were calculated within each SST bin of 0.3°C.

Generalized Additive Models (GAM) were also used to explore the relationship between sardine-NASC and the different variables. A geometric average of sardine-NASC was used because of the log-normal distribution of the sardine abundance. The GAMs analyses were performed using the ‘‘mgcv” library [41] for the “R” statistics software [42]. GAMs help reveal possible functional or predictor-response relationships using non-parametric models. The GAMs use a link function to establish a relationship between the mean of the response variable and a ‘‘smoothed” function of the explanatory variable(s). They are useful when the relationship between variables is expected to be complex and not easily fit to standard linear or non-linear models [43]. The GAM analyses were performed with a Gaussian distribution, applied with the assumption that sardine-NASC is a continuous variable with a log-normal distribution. Output plots from GAMs illustrated the non-linear relationship between the response (sardine-NASC) variable and each predictor (SST, SST-gradient, distance to the nearest upwelling focus, Chl, and location). Location was tested as a predictor for the geographical distribution of sardine-NASC by implementing a two-dimensional smoothing filter [44] of the sample geographic coordinates (Lon, Lat).

## 3. Results

### 3.1 Acoustic surveys

The spatially-averaged SST during each of the VECEP cruises was well within or around one standard deviation of the long-term SST climatology (1994–2009, Fig 4). Because of this, although the surveys were done in different years, we combined all data as if they were part of a typical annual cycle.

**Fig 4:**
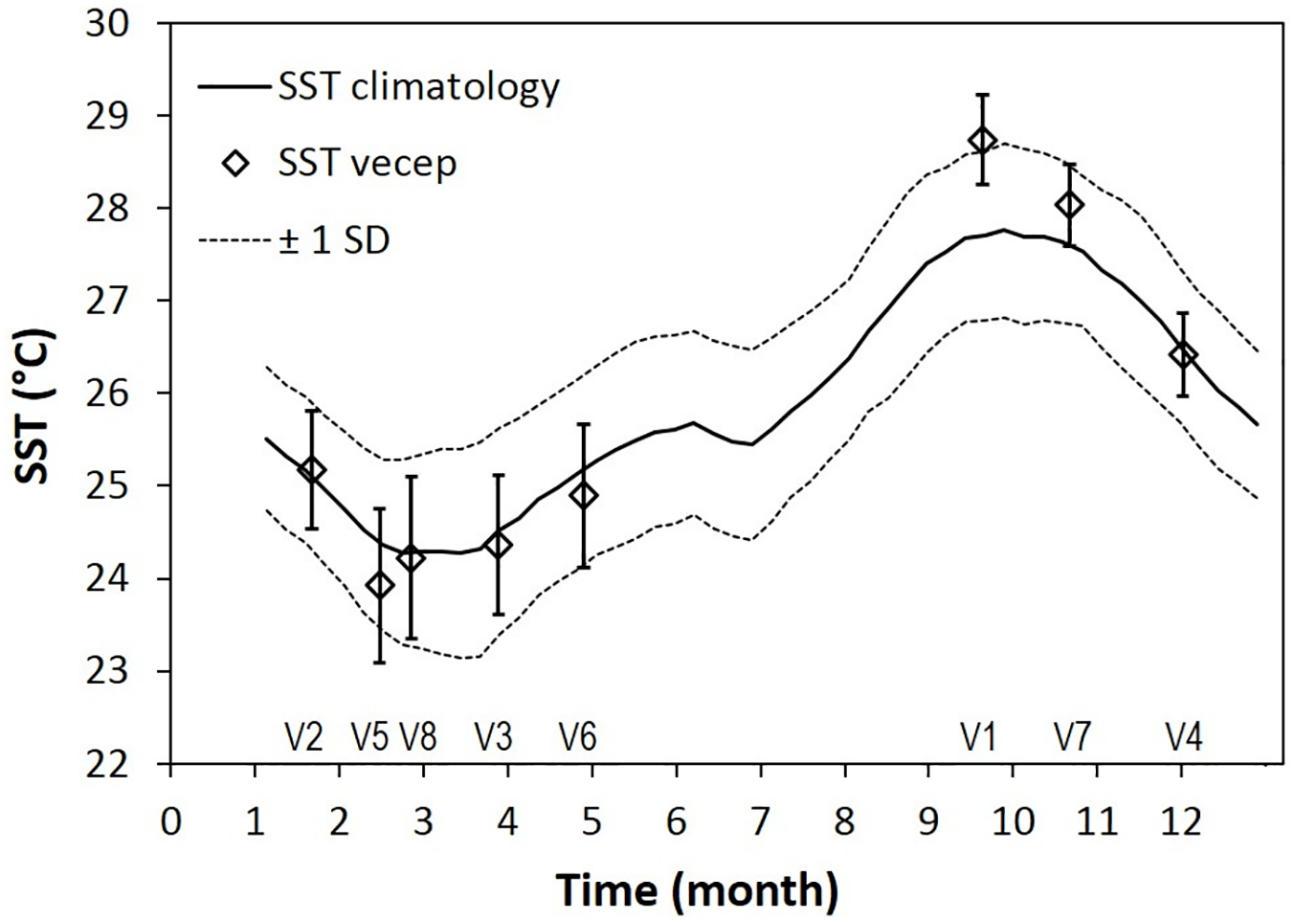
Spatially-averaged SST during each VECEP cruise compared with the SST climatology. Spatially-averaged SST during each VECEP acoustic cruise (V1 to V8) compared to the weekly SST climatology (1994–2009). Spatial averages and standard deviations (SD) were calculated for the area defined in Fig 2C, which excludes the Orinoco plume. An envelope of ±1 SD are shown for the climatology (dotted line) and the Vecep averages (bars).

**Fig 5:**
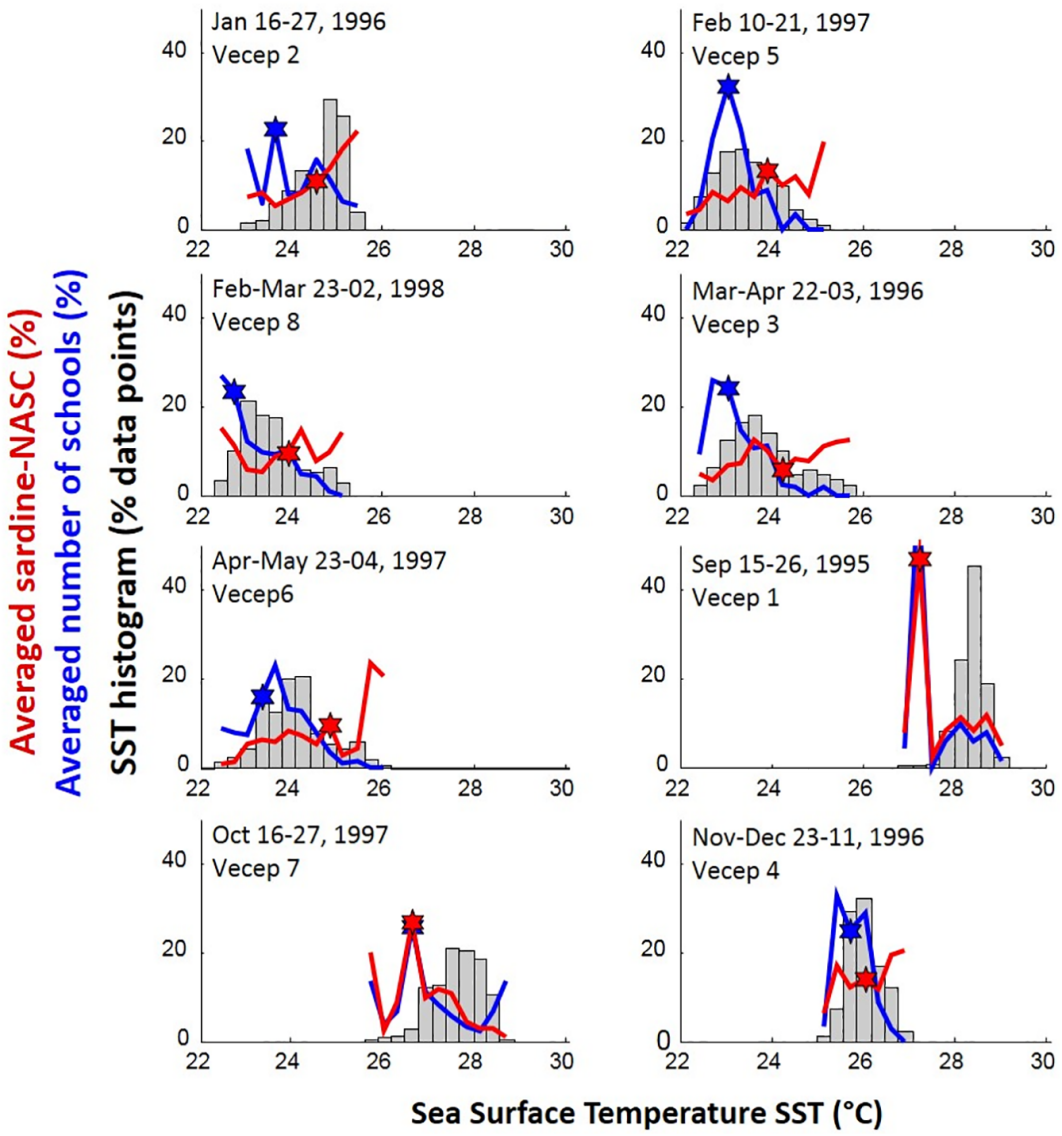
Averages of sardine-NASC and schools for SST intervals. SST histograms calculated from SST extracted along each cruise track. For each SST-bin of 0.3°C, averages of log(sardine-NASC) (red line) and of the number of small pelagic schools (blue line) were calculated and scaled to percent. Stars indicate the SST-bin where 50% of the average sardine-NASC and school number occurred.

As expected, the Spanish sardine relative abundance index showed strong patchiness (aggregative behavior) without an obvious relationship to SST (Fig 3). However, the sardine-NASC abundance showed a seasonal pattern when averaged within each bin of the SST histograms (Fig 5). During the upwelling season (December to May) sardine-NASC was found dispersed throughout the SST range. This indicated a wide spatial distribution of sardine-NASC patches in the study area. On the other hand, during the period of weak upwelling (September and October surveys) sardine-NASC was higher in the coolest SSTs (Fig 5). During these warm months, cooler SSTs were found very close to the coast, where upwelled waters reach closest to the surface. The most evident coastal upwelling foci during September-October were seen along the Araya and Paria Peninsulas, and within the Gulf of Cariaco (Fig 3), and sardine-NASC was highest in those areas. In contrast to sardine-NASC clear seasonality, the number of small pelagic schools was always higher in the cooler SSTs during all the surveys (Fig 5).

**Fig 6:**
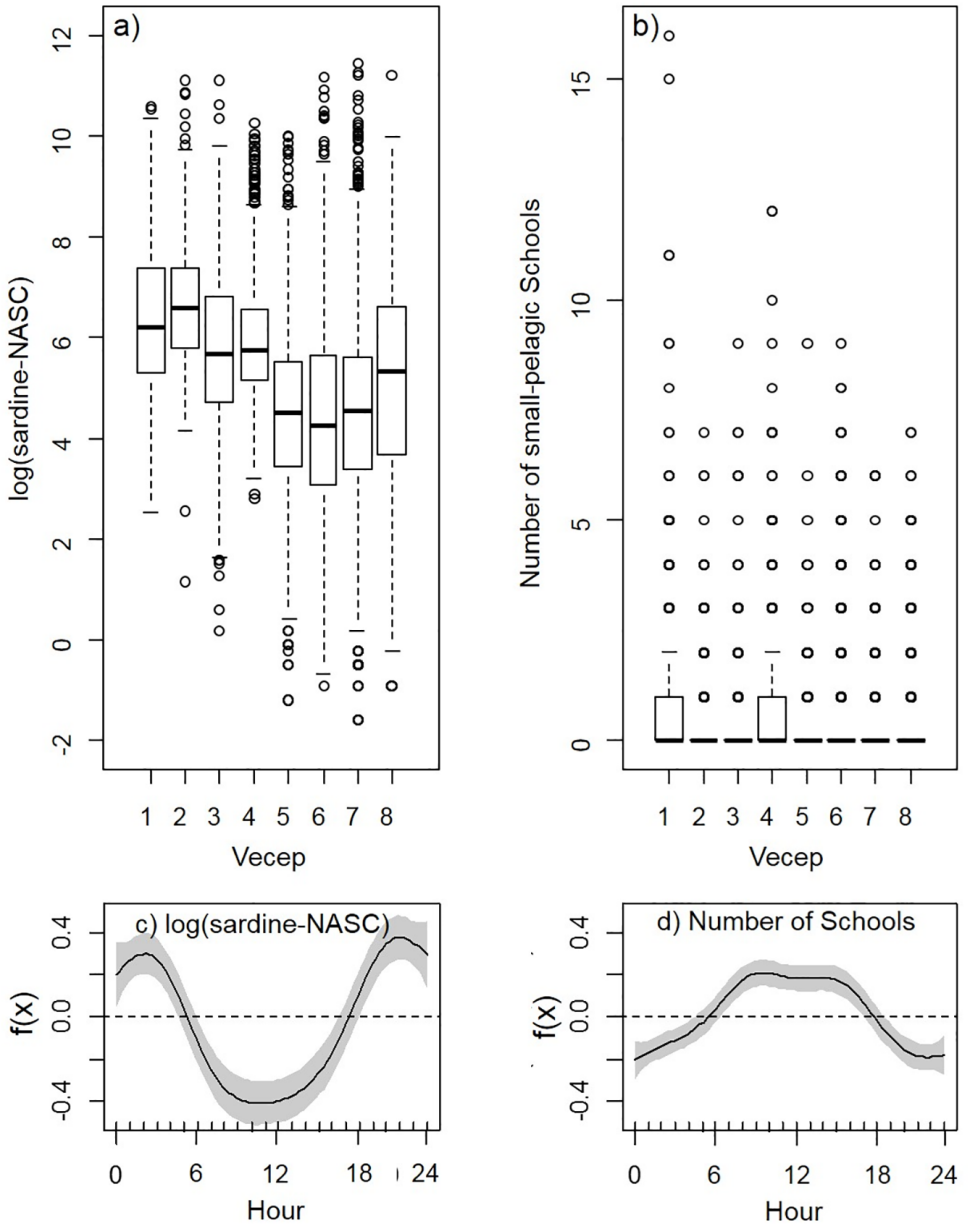
Boxplots and Generalized Additive Model (GAM) analysis of sardine-NASC and Schools for each Vecep cruise. Boxplots of (a) sardine-NASC and (b) Schools for each Vecep cruise, and GAM analysis of (c) log(sardine-NASC) and (d) number of Schools vs. hour. Regardless of the season, the first four surveys showed higher averages of sardine-NASC than the last four surveys; however, the number of schools didn’t show differences between surveys. The GAM analyses show the typical diel patterns of aggregation of sardines. The backscattering strength of sardine is weaker during day time, because they are concentrated into school of fish; the opposite occurs during night time.

Regardless of season, the first four VECEP surveys (September 1995; and January, March, and November-December 1996) had significantly higher sardine-NASC biomass and lower variances than the last four surveys (February, April, and October 1997; and February-March 1998; see Figs 3 and 6A; ANOVA, p<0.01). Yet, the number of pelagic Schools did not show differences between surveys (Fig 6B). The spatial averages of SST during those VECEP surveys were well within ± 1 standard deviation of the long term SST average for that area (Fig 4). However, other external conditions that may affect the sardine habitat cannot be discarded, such as a late or short upwelling season, low primary production, changes in currents, changes in fishery pressure, etc. Previous hydroacoustics surveys in the area also show inter-survey variability that could not be explained easily [7].

The backscattering strength of sardine-NASC density showed expected diel variations during all surveys, with higher backscatter recorded during nighttime (between 5:30 PM and 5:30 AM, Fig 6C; ANOVA p<0.01). The number of Schools exhibited the opposite diel pattern, with higher degree of aggregation during daytime (Fig 6D). This diel pattern, with small pelagics dispersing during the night and re-forming schools during the day is common [45, 46]. For the quantification of sardine biomass it has been recommended the use of nocturnal data, when sardine aggregations are less dense, with a more homogenous spatial distribution, and less tendency of avoidance [7, 47, 48]. Cárdenas and Achury [6] compared the mean abundance, dispersion, and coefficient of variation for each VECEP cruise; they found no significant differences between day and night measurements, and therefore, they used all day/night data in their analysis. Our approach was to use the whole day/night dataset for the Generalized Additive Models (GAM) analysis, but taking into account the influence of the diel cycle on the sardine-NASC and on the pelagic school number (Fig 6C and 6D) by including the variable ‘sampling hour’ as a categorical main factor in the model. Following the same reasoning, we also included as main categorical factors the variable ‘survey number’ to account for inter-cruises variability (Fig 6A), and the variable ‘area’ to account for variability on the sardine biomass conversion factors calculated for the three exploratory fishing areas (Fig 1B, section 2.2). An example of the GAM model including the influence of these variables is shown in Eq (2). The inclusion of the main categorical factors improved the fit of the models.

GAM analyses can be applied to several independent variables at the same time (multivariate model) to obtain the best model that explain the variations of the dependent variable. However, concurvity (non-linear multicollinearity) could produce unstable or inaccurate estimates of the covariates’ functional effects [49, 50]. Concurvity occurs when some term in a model can be approximated by one or more of the other smooth terms in the model. We calculated the level of concurvity between the predictors and used the ‘estimate’ index (values between 0 and 1, with 0 indicating no problem, and 1 indicating total lack of identifiability [51]). The data used in this study had high levels of concurvity with Location (SST 0.78, Chl 0.79, upw-foci 0.91); and from low to moderate concurvity (SST 0.32, Chl 0.20) with distance to the nearest upwelling foci, and with SST (Chl 0.27). High concurvity was due to the spatial stability of both the upwelling foci and the upwelling plumes, and also due to the correlation between upwelling and phytoplankton biomass (Fig 2C).

The influence of some predictors can change when analyzed together or separately. For example, Jablonski and Legey [52] found an optimal window dome-shaped relationship between recruitment with Ekman Transport and with SST when used separately, but the dome shape was lost when the terms were used together. Since our objective was to understand the relationship of sardine-NASC and Schools with the different variables, and in order to avoid the problems produced by concurvity, we analyzed univariate GAM models with one parameter at a time (SST, upwelling foci distance, Location and Chl; see for example Bertrand et al. [53]). Each model included the main categorical factors of survey sampling time (hour) and number (survey) for sardine-NASC and Schools, and also exploratory fishing area (area) for sardine-NASC. An example of a univariate GAM formula for SST is given below:

GAM(log(sardine-NASC))∼s(SST)+factor(hour)+factor(survey)+factor(area),data=Veceps (2)

To contrast the differences in sardine-NASC distribution during different phases of the seasonal upwelling cycle, we analyzed three separate seasons (see Figs 3 and 4): (a) the strong upwelling season (i.e., ‘cool’ conditions, between January-April) included VECEP cruises 2, 3, 5, 6 and 8. (b) the weak upwelling season (i.e., ‘warm’ conditions, when the seasonal SST maximum is reached between September-October) used VECEP 1 and 7. (c) upwelling transition (‘transition’ conditions, from August to November), was analyzed using VECEP 4.

**Fig 7:**
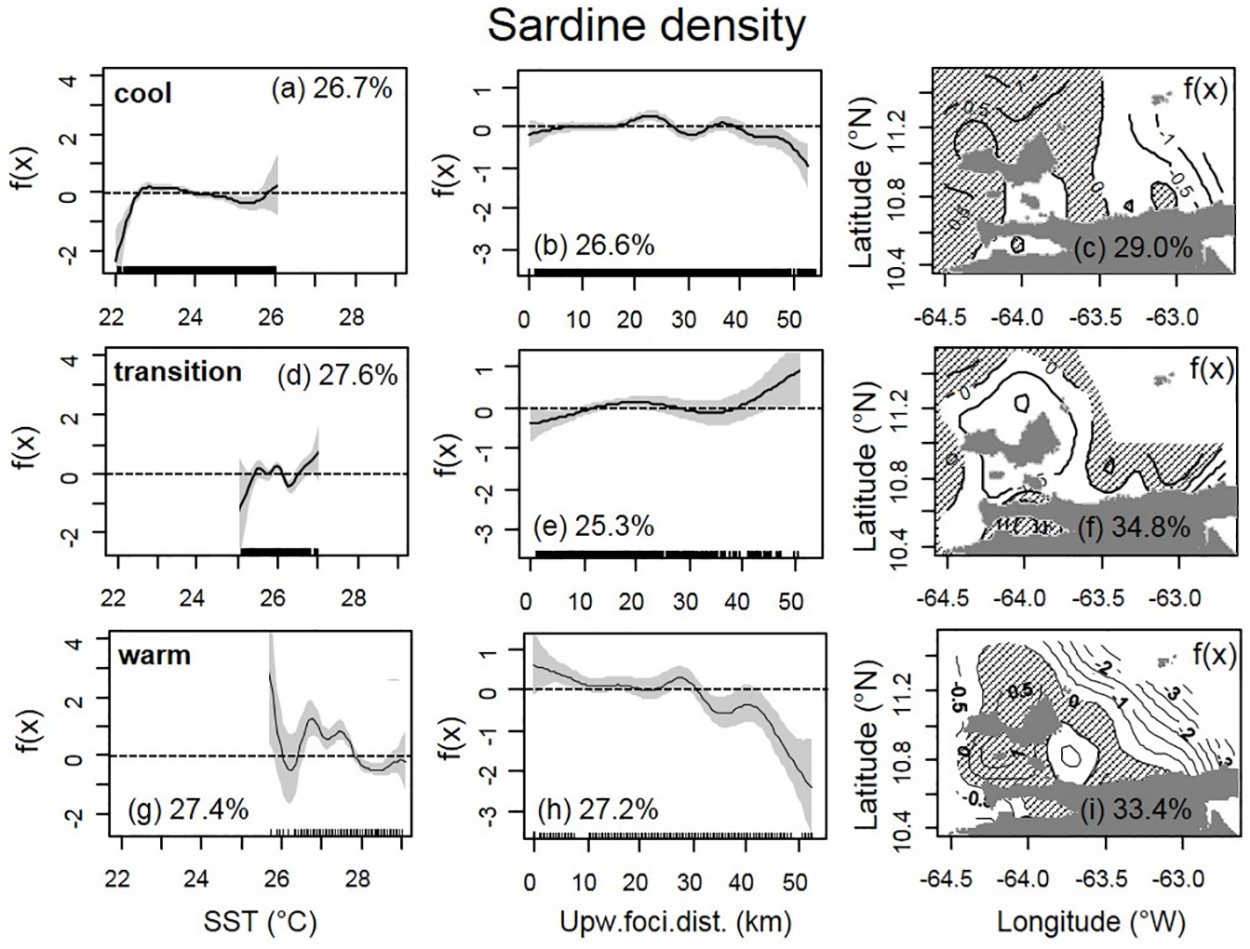
GAM analysis of sardine-NASC *vs*. SST, distance from upwelling foci, and location for different seasons. Univariate GAM analysis of log(sardine-NASC) *vs*. SST, distance from upwelling foci, and Location (longitude, latitude) for: Cool (top a, b and c, VECEP 2, 3, 5, 6 and 8), Transition (middle d, e and f, VECEP 4), and “Warm” (bottom g, h and i, VECEP 1 and 7) conditions. The y-axes (and the isolines in the map) show relative changes of the environmental variable on sardine-NASC, f(x), so that a value of zero represents the mean effect. Positive isolines in Location are highlighted with hashed lines. Each parameter has similar scales to contrast its importance between different upwelling conditions. For SST and foci distance the values where the data lay is marked on the x-axis (rugplot). The 95% confidence intervals are grey-shaded for SST and foci distance. All terms were significant (p<0.01) and the sardine-NASC ‘percentage of deviance explained’ (analogous to variance in a linear regression) are shown for each term.

Output plots from GAMs illustrate the non-linear relationship between the response variable and each predictor. For example, in Fig 7A the y-axis represents a relative scale with zero as the mean effect of the independent variable or predictor (i.e., SST) on the response variable (i.e., log(sardine-NASC)), and positive/negative y-values indicate a positive/negative effect on the response. The range of the function in the y-axis indicates the relative importance of each predictor. The black highlight marks on the x-axis (the rugplot) indicate where the data values lie. GAM graphs show the 95% confidence limits (grey envelope). These limits often diverge near the extreme of the range for continuous predictors as a consequence of fewer observations.

**Fig 8:**
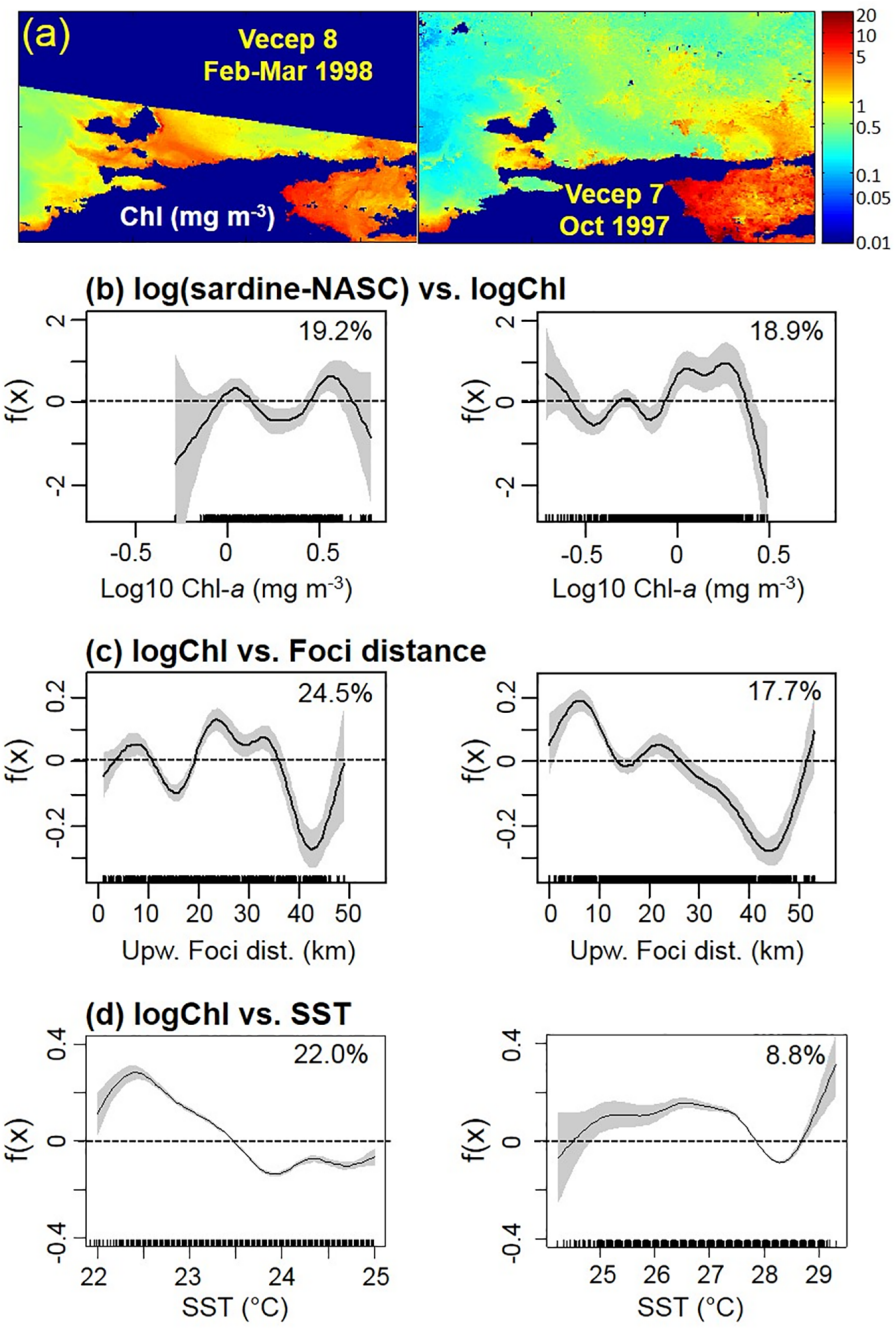
Chlorophyll-a conditions and GAM analysis between strong and weak upwelling conditions. Comparison of Chlorophyll-a conditions between strong upwelling (left column) and weak upwelling (right column) conditions for: (a) Satellite Chlorophyll-a (Chl); GAM analysis of (b) log(sardine-NASC) vs. logChl, (c) logChl vs. distance from upwelling foci, and (d) logChl vs. SST. GAM 95% confidence intervals are grey shaded. All terms were significant (p<0.01) and the percentage of deviance explained are shown.

During ‘cool conditions’ (temperature range of 22–26°C; Fig 7A) sardine-NASC was distributed evenly across the temperature range for SST > 22.5°C, but it was smaller at cooler temperatures. Log-transformed satellite Chl for February-Mar 1998 (VECEP 8) had an inverse linear correlation with SST between 22.4°C and 24°C (Fig 8D), due to the higher nutrient content of the cooler upwelled waters. However, for waters cooler than 22.4°C the logChl-SST relationship turned direct (Fig 8D), with less phytoplankton in the coolest SSTs. This direct SST-Chl relation is characteristic of newly upwelled waters [54, 55]. Basically the GAM results for sardine-NASC were negative within the coolest waters of the upwelling season (Fig 7A) due to plankton scarcity (Fig 8D). During this period, distance to upwelling foci did not show any influence on sardine-NASC (Fig 7B) because the sardine was widely distributed over the study area, especially north of 11°N (Fig 7C). Slightly lower sardine concentrations occurred in the Gulf of Cariaco and between the Margarita Island and the Araya Peninsula. Sardines showed a slight avoidance for the eastern region (Fig 7C), which may be related to the Orinoco discharge plume. During VECEP 8, cooler upwelled waters (Fig 3) and high Chl (Fig 8A) were widely spread over the continental shelf. The highest Chl was found 20–35 km from the upwelling foci (Fig 8C). Biomass of sardine-NASC showed two peaks in relation to Chl concentration (around Chl 1.1 and 4 mg m^-3^, or logChl 0.05 and 0.6; Fig 8B).

During ‘transition conditions’ (temperature range of 25.1–27°C; Fig 7D), high sardine biomass also occurred in the entire study area (Fig 7E and 7F), with the highest sardine-NASC within the Gulf of Cariaco (Fig 7F) and some apparent avoidance of waters in the immediate vicinity of Margarita Island. There was preference for intermediate SST (25.4–26.1°C, Fig 7D).

During ‘warm’ conditions (range of 25.7–29.1°C; Fig 7G) sardine-NASC showed a strong avoidance of the area of the Orinoco River plume (Fig 7I, Fig 2C). The cruises of September and October occurred during the season of highest Orinoco discharge water into the southeast Caribbean Sea (form July to October [25]). Sardines were concentrated in the coolest SSTs (26.3–27.7°C) closer to the coast within 10 km of the upwelling foci, and showed strong avoidance of areas farther than 32 km from the coast (Fig 7G and 7H). The cruise VECEP 7, carried out during the warm season, showed focal upwelling very close to the coast, and with higher Chl (Fig 3 and Fig 8A). During VECEP 7, sardine-NASC was higher where Chl concentrations were higher (1–2.2 mg m^-3^, or 0–0.35 logChl, Fig 8B) near to the upwelling foci (i.e. within 8 km, Fig 8C). The high Chl in the warmest SST >29°C (Fig 8C and 8D) were due to CDOM contamination from the Orinoco plume (Fig 8A).

The GAM sardine-NASC percentage deviance explained (analogous to variance in a linear regression) during the three upwelling seasons was always higher with the spatial location (Lon, Lat) than with SST or upw-foci (Table 1, Figs 7 and 8). This is due to the fixed location of the upwelling foci and due to the high spatial stability shown by the seasonal dispersion of the upwelling plumes and of the Orinoco River plume. There were no significant improvements on the percentage of deviance explained using a univariate model with only spatial location as the ‘‘smoothed” function of the explanatory variable, or using a multivariate model (i.e. comparing sardine-NASC with the spatial location together with SST or upw-foci or both, Table 1).

**Table 1:** GAM’s percentage of deviance explained of sardine-NASC and Schools with the different environmental predictors for the three seasons of the upwelling cycle.

		Cool				Transition				Warm			
		Lon-Lat	SST	Foci	All	Lon-Lat	SST	Foci	All	Lon-Lat	SST	Foci	All
NASC	Lon-Lat	**29.1**				**34.8**				**33.4**			
	SST	29.6	**26.7**			36.3	**27.6**			34.9	**27.4**		
	Foci	29.3	27.2	**26.6**		35.5	28.5	**25.3**		34.8	29.3	**27.2**	
	All			** **	***29*.*8***	** **	** **	** **	***36*.*5***	** **	** **	** **	***36*.*3***
Schools	Lon-Lat	**29**				**31.4**				**24.5**			
	SST	30	**27.2**			31.9	**20.1**			25.2	**17.3**		
	Foci	29.8	27.5	**26.5**		31.5	20.4	**15.8**		25.2	21.4	**18.9**	
	All			** **	***30*.*8***	*** ***	*** ***	*** ***	***32*.*0***	** **	** **	** **	***26*.*7***

GAM’s percentage of deviance explained is analogous to variance in a linear regression. The environmental predictors used were location (Lon-Lat), sea surface temperature (SST), and distance from upwelling foci (Foci). GAM was performed for univariate models (i.e. comparing sardine-NASC with each predictor separated, in bold), for bivariate models (i.e. comparing sardine-NASC with the pair of predictors placed in the column and row), and multivariate model with the three predictors together (highlighted in bold and italics).

**Fig 9:**
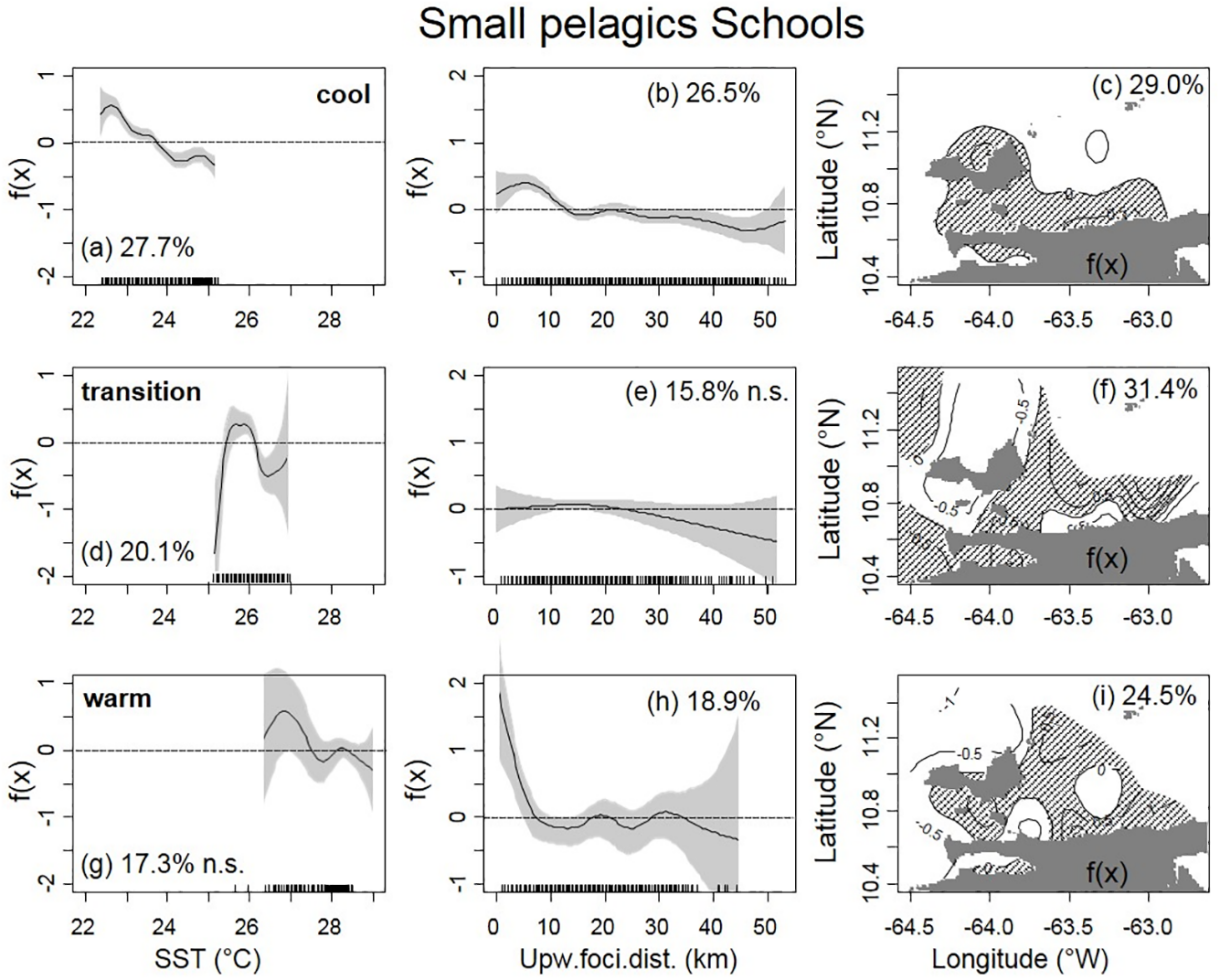
GAM analysis of schools *vs*. SST, distance from upwelling foci, and location for different seasons. Univariate GAM analysis of the number of small pelagics schools vs. SST, distance from upwelling foci, and location (longitude, latitude) for: Cool (top a, b and c, VECEP 2, 3, 5, 6 and 8), Transition (middle d, e and f, VECEP 4) and weak or “Warm” (bottom g, h and i, VECEP 1 and 7) upwelling conditions. GAM 95% confidence intervals are grey shaded; Lat-Lon positive isolines are highlighted with hashed lines. Percentage of deviance explained is shown for each term. All terms were significant (p<0.01), except for 9e and 9g, which were no-significant (n.s.).

Similarly to the histogram analysis, GAM results showed higher number of small pelagic schools (Schools) in the cooler SST range (Figs 5 and 9). The number of Schools was higher within 10 km of upwelling foci during “cool” and, especially, during “warm” conditions (Fig 9B and 9H). For “transition” conditions, there were fewer Schools at distances >30 km from the upwelling foci (Fig 9E). Higher number of Schools were present closer to the coast for all the seasons, although in “transition” conditions there was a tendency to have a higher number of Schools in offshore waters (Fig 9C, 9F and 9I). Similarly to sardine-NASC, the Schools percentage of deviance explained by the spatial location alone was higher than with SST or with upw-foci alone, and the bivariate and multivariate models didn’t improve significantly those values (Table 1).

The hour of the day was included as a categorical main factor to the GAM analysis, and the GAM summary showed the hour effect on the response variable. For the number of pelagic schools, during “cool” conditions the significant time period with higher number of schools was from 6 am to 8 pm; while during “warm” conditions that period was shorter, from 5 am to 5 pm. GAM analysis of the SST-gradient effects on sardine-NASC and Schools did not show any significance for any of the upwelling conditions (p-values of SST-gradient vs. NASC / Schools were 0.06 / 0.58, 0.24 / 0.70, 0.31 / 0.70, for cool, transition, and warm conditions, respectively).

**Fig 10:**
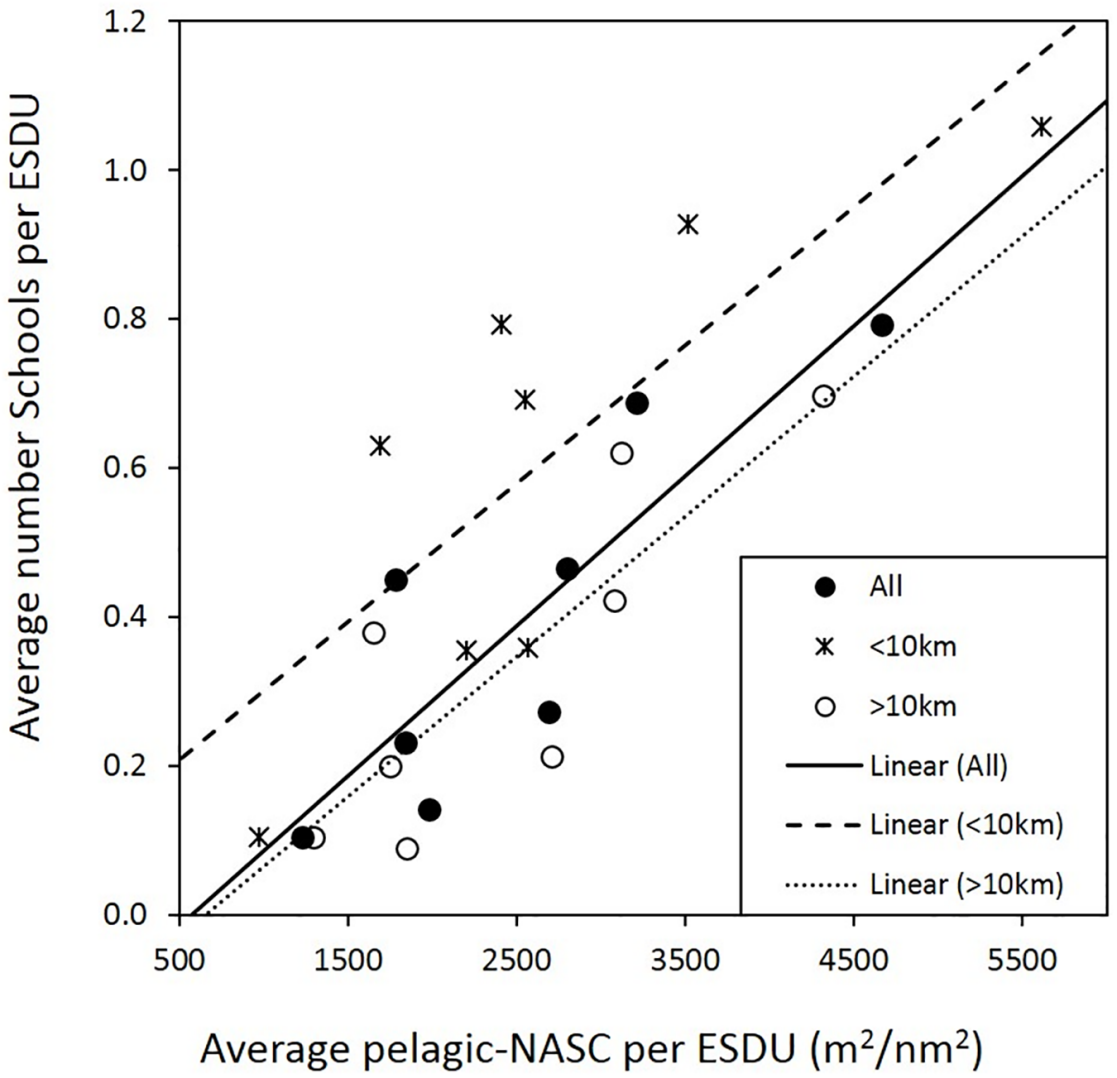
Relationship between the pelagic fish density and the number of pelagic school averaged for each VECEP cruise. Pelagic fish density (pelagic-NASC) vs the number of pelagic schools averaged for all the elementary sampling distance unit (ESDU) at each cruise (All), and for the ESDUs within 10 km of the upwelling foci (<10km) and the ESDUs farther offshore (>10km). Correlations were significant (p<0.05) and with similar slope, but data closer to the upwelling foci had a higher intercept, due to the larger average of school number closer to the coast in comparison with the rest of the ESDUs.

There was no apparent correlation between the number of pelagic schools with pelagic-NASC or with sardine-NASC when we compared the ESDU values directly. This is a result of the highly spatial-temporal variability of the dataset. However, when comparing the cruise averages for these variables, a direct correlation between biomass and the number of schools was discernible (Fig 10). Averaged pelagic-NASC had significant correlations with averaged pelagic schools for all the data set, and also for the ESDUs within 10 km from the upwelling foci, and for the ESDUs farther offshore (Fig 10, n = 8, R^2^ of 0.74, 0.64, and 0.71 at p<0.01, <0.05, and <0.01, respectively). Those three correlations had similar rate of change on the number of schools per NASC (no differences between the slopes, p>0.1); however, ESDUs closer to the upwelling foci had higher number of pelagic schools per biomass (significant differences in the intercepts, p<0.05, Fig 10). This is another evidence for the higher proportion of schools closer to the coast during all the seasons sampled, compared with offshore values. In the other hand, correlation of the average of pelagic schools with the average sardine-NASC was only significant for data within the first 10 km of the upwelling foci (n = 8, R^2^ = 0.37, p<0.1). This is expected, as not all the pelagic school detected were from *S*. *aurita*. When using the total sum of the pelagic-NASC and of the pelagic schools per cruise—instead of the averages—, correlations were much weaker (n = 8, all data: R^2^ 0.46, p<0.1; data within <10km from upwelling foci: R^2^ 0.53, p<0.05; data offshore >10km: R^2^ 0.27, p>0.1); and for the total sum of the sardine-NASC all correlations were no significant.

### 3.2 Annual averages of sardine catch, SST and CHL

**Fig 11:**
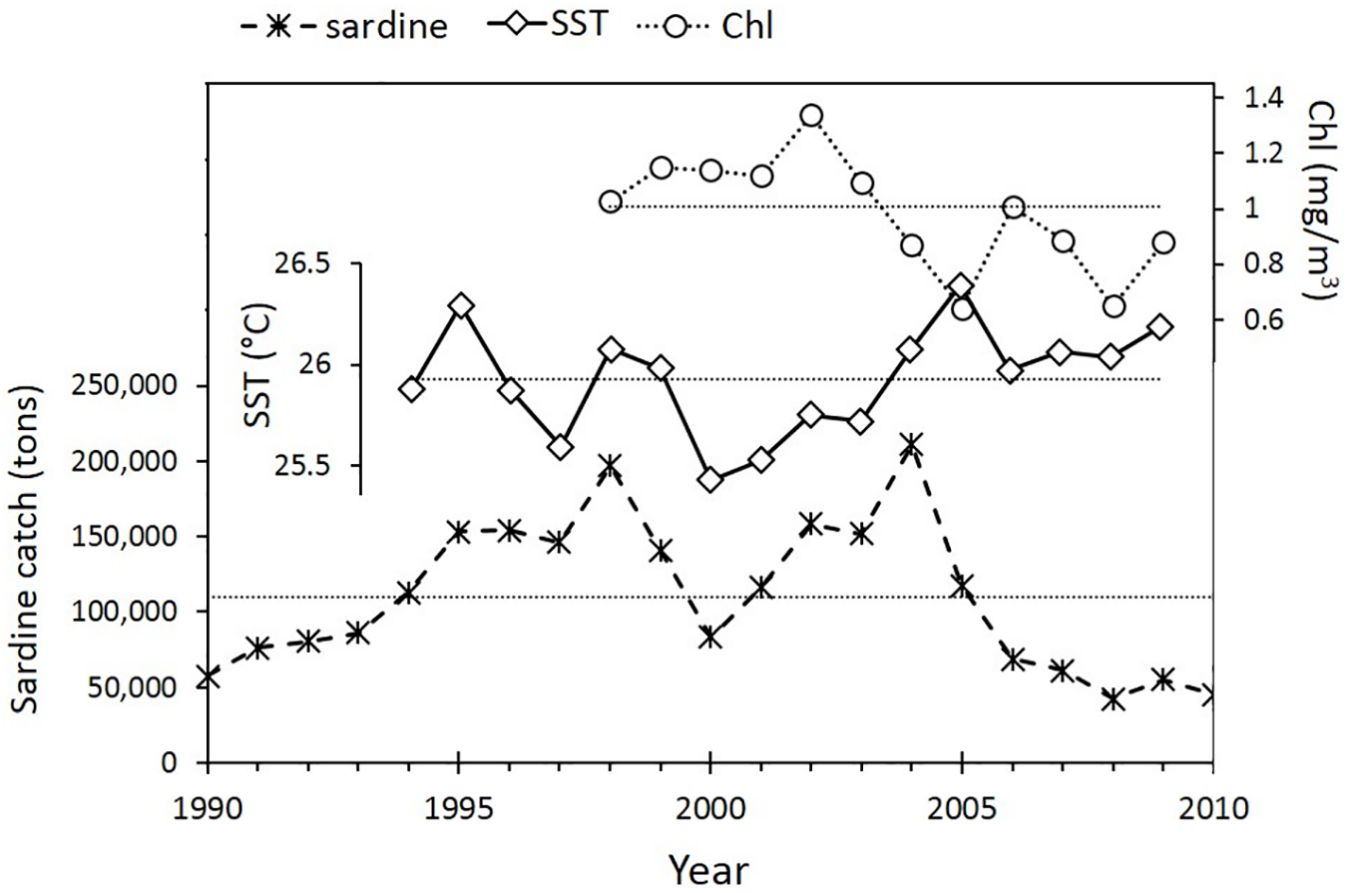
Comparison of annual catch of *Sardinella aurita* in northeastern Venezuela with annual spatial averages of SST and Chl. SST and Chl were spatially-averaged for the area demarked in Fig 2C. Long term means of sardine catch (1990–2010), SST (1994–2009) and Chl (1998–2009) are shown for reference (horizontal dotted lines). Annual catch of Spanish sardine was obtained from the Global Capture Production Dataset from FAO [4]. Up to 2014 the annual sardine catch had remained around the 2010 catch of 50,000 tons [4].

The average of *Sardinella aurita* landings off northeastern Venezuela for 1990–2010 was 110,268 tons (Fig 11). Spanish sardine landings showed marked increases during two years of weak upwelling, namely 1998 and 2004 (196,798 and 211,092 tons, respectively), but these values declined abruptly the following years (Fig 11). After the warm upwelling anomalies of 1998–1999, the 2000–2003 period had intense upwelling. Sardine landings started to increase in 2001, one year after the first year of intense upwelling. On the other hand, after the peak landing of 2004, the year 2005 was even warmer and showed very low chlorophyll, but still featured relatively high sardine landings (117,800 tons). Afterward, warmer than average conditions continued for several years, also with lower than average chlorophyll (Fig 11). Catches then declined to less than 20% the landings in 2004 (Fig 11), and as of 2014 (the last year in record at FAO [4]) the fisheries had still not recovered.

## 4. Discussion

### 4.1 Spatial biomass distribution related to the upwelling cycle

The seasonal differences in the spatial distribution of Spanish sardine in the Southeastern Caribbean Sea were closely related to changes in their habitat due to the spatial distribution of upwelled waters and associated phytoplankton biomass. This agrees with the previous analyses of the VECEP series [6]. Our analysis, however, bring a higher level of details on the sardine distribution. During strong upwelling conditions the upwelling plumes are large, and Spanish sardine is also widely distributed because it can find food over a larger and broader area. However, during the warm season (September-October) Spanish sardine biomass and schools tended to concentrate in the first 10 km from the upwelling foci, where waters are cooler and with higher phytoplankton concentrations than the surrounding waters, due to localized upwelling. During the warm season the favorable habitat of the Spanish sardine is greatly reduced to waters closer to the coast, where the upwelling process is limited to few kilometers offshore the coast, near the upwelling foci locations [2, 19].

When Spanish sardine biomass and schools are closest to the coast during the warm season, the stock becomes more accessible to the artisanal fishery that operates within 10 km offshore. This is supported by the seasonality shown by the Catch Per Unit eEfort (CPUE) of this stock, with lower CPUE during the principal upwelling (December-April) and higher CPUE during the rest of the year (averages for 1973–1989, [56]). Additionally, the reproductive index of this stock starts to increase in September [31]; therefore, a growing proportion of Spanish sardine with mature gonads would be closer to the coast and more available to the fishery. Further ecological pressure is produced when the spatial range of this species shrinks during the warm season, due to increased competition for food and higher levels of cannibalism of eggs and larvae [57].

*Sardinella aurita* showed strong avoidance of the Orinoco River plume during September-October. No significant numbers of eggs and larvae have been found to the east of this front of fresher and warmer waters [58] because this specie is stenohaline [59, 60]. The maximum discharge of the Orinoco Rivers occurs from July to October [25]. Consequently, during the warmest SST season in September-October there are two mechanisms that influence the contraction of *S*. *aurita* to a smaller area: (a) localized cells of upwelling very close to the coast, that induce Spanish sardine concentration closer to shore; and (b) a wider hypohaline front due to the discharge of the Orinoco River to the east, that causes a horizontal migration of the stock away from this front. A completely different scenario might occur during July, when the maximum freshwater extension of the Orinoco River coincides with the mid-year upwelling [19]. Unfortunately, the acoustic VECEP series did not have any cruises during that time frame.

The largest deviance for every VECEP survey was explained by geographic location (Table 1). The same has been found for herring and sprat in the Gulf of Finland [61]. This is the result of the fixed geographic location of the upwelling foci, the stable dispersion of the upwelling plumes, and of the geographic features that lead to enhanced upwelling and chlorophyll in certain sectors, which ultimately influence the distribution of Spanish sardine. Other geographically related factors not studied here could also have influence on small pelagics distribution, such as bathymetry, seabed type, salinity and the continental platform dynamics [62–67].

During the principal upwelling season, *Sardinella aurita* showed a strong avoidance to the coolest temperatures (SST < 22.5°C, Fig 7A), which has been documented for small pelagic fish in other areas [65, 68, 69]. Zwolinski et al. [69] suggest that the reason for the apparent preference for mid-range SST could be inadequate food for adults in the cooler freshly upwelled coastal waters. In northeastern Venezuela, the adult of *S*. *aurita* is an opportunistic feeder and it feeds largely on phytoplankton (Cellamare and Gómez [67] and references therein). Off northeastern Venezuela, the positive effect of SST on sardine biomass started at 22.5°C (Fig 7A), coinciding with the SST with a maximum chlorophyll peak during upwelling conditions (Fig 8D); however, at cooler temperatures chlorophyll was still higher than the mean. Therefore, sardine avoidance of the coolest waters was not due to shortage of phytoplankton per se, and it may be related to the quality of the food [69] or due to physiological preferences (*S*. *aurita* is considered a tropical stenothermic species [70]).

Higher abundance of small pelagics along thermal fronts has been found in the North Sea [71] and northern Chile [72]. However, we did not find any significant effect of thermal gradients on sardine biomass or on school number.

### 4.2 Spatial distribution of schools

Exploratory fishing presented by Cárdenas and Achury [6] found that more than half of the small pelagic schools from the VECEP cruises were *Sardinella aurita*. School number was directly related to the pelagic-fish density (Fig 10), but their relation with the sardine density was weak. This is expected, as not all the pelagic schools detected were from *S*. *aurita*.

Regardless of the upwelling season, all surveys had higher numbers of small pelagic schools in the cooler waters closer to the coast, and this was especially pronounced during warm upwelling conditions (e.g. Figs 5 and 9). This preference for nearshore schooling is also evident for other 6 acoustic surveys on the same study area carried out between 1985 and 1989 [7]. Schooling has a multi-purpose function related to survival of predator attacks, effectiveness of feeding, hydrodynamic swimming advantages, migration, reproduction, social learning, etc. [73, 74, 75, 76]. The number of school for ESDU may relate biologically to occupation of the habitat. In the study area, the higher proportion of pelagic schools for the coastal cooler waters available (and hence, in waters with higher levels of phytoplankton) suggests that higher number of schools in this specific habitat might be related to higher food availability. Waters with higher levels of plankton would ensure lower levels of intra-school food competition [9]. During the “cool” season, the diel pattern of higher school numbers was two hours more prolonged than during the “warm” season. This might indicate that during periods of higher productivity due to upwelling, the habitat is capable to sustain small pelagic schooling during a prolonged time. There is a relation between pelagic fish biomass and the number of schools in the study area (Fig 10). Nevertheless, when compared to offshore data, higher proportion of schools were found by unit of fish density closer to the upwelling foci (Fig 10). This is another evidence of the higher proportion of schools closer to the coast year-round, regardless of the season. Due to the lack of information on the dimension or density of the schools, we cannot evaluate spatial or seasonal differences on those school characteristics. However, it has been found that declining fish populations are distributed among fewer schools, but not smaller schools [77], in other words, school sizes do not change with stock biomass.

A higher proportion of pelagic schools in northeastern Venezuela are closer to the coast within the reach of the artisanal fishery year-round. The number of schools is directly proportional to the fish abundance (Fig 10, [77]) and during warm conditions a higher proportion of sardine-NASC tends to be located closer to the coast (Fig 7). This implies that during years with general diminished sardine biomass, a higher proportion of that biomass–and schools–will be closer to the coast during the warm season available to the fishery. With a general lower biomass the number of school would be less, but their size would not change [77]. Since school characteristics affect catchability, the ability of fishermen to locate these schools remains high, and catches and catch rates are maintained despite reduced abundance. As a result, catch rates would not decrease dramatically when the stock declines, until the reduced abundance of schools begins to impact fishing success or the stock even collapses [73].

### 4.3 Significance of the results for the Spanish sardine fishery

The seasonal behavior of coastal concentration of *S*. *aurita* biomass and schools during the season with warmest SST, might be more prolonged during years with anomalous weak upwelling. Our results can be used to interpret the collapse of the Spanish sardine fishery observed in the southeastern Caribbean region in 1998 and 2004. The two highest reported annual sardine catches of the last three decades co-occurred with two years of warm SST (1998 and 2004, Fig 11). During years with weak upwelling there would be prolonged food scarcity, and also increased catchability due to both biomass migrations toward the coast and the around-the-year higher proportion of schools closer to the coast. This “perfect-storm” scenario would severely affect stocks if overfishing is sustained for consecutive years of weak upwelling. The sardine fishery indeed plummeted after the catch peak of 1998 but recovered quickly by 2001, after one year of strong upwelling in 2000 (Fig 11). The recruitment of the Venezuelan stock of *S*. *aurita* is continuous in time, and it occurs before the first year of age with a size of 15–17 cm [31, 78]. This fast recruitment explains the recuperation of the fishery one year after a year with strong upwelling conditions. However, the high catch, low-upwelling year of 2004 was followed by several even warmer years with lower Chl in the study area up to 2010 (Fig 11), which didn’t allow the recuperation of the stock. This substantial warming and decreased upwelling has been reported for the Southeastern Caribbean Sea since 2004 [79].

The Spanish sardine was most likely overfished in 2005, a warm year with likely high levels of unreported fishing. This was a period when the sardine commanded very high prices in US dollars in the Caribbean market outside Venezuela. There are many undocumented reports of fish catch from Venezuelan coastal waters being sold abroad. These catches were not reported in Venezuela. Consecutive years of weak upwelling did not allow the sardine population to recover, and in 2014, the catch was still <25% the official capture levels seen in 2004 [4]. Hydroacoustic stock surveys done in this area in October 2009 showed a biomass reduction of this stock by 70–90% compared to the average of the previous three decades [15].

Under adverse conditions the Peruvian anchoveta, experiences drastic declines and may collapse completely due partly through reduced food supply, and partly through enhanced vulnerability to fishing pressure caused by aggregation of the fish in the remaining foraging areas [80]. The Venezuelan *S*. *aurita* stock has a short longevity (A_0.95_ = 3.3 years [74, 81, 82]), similar to the longevity of about 3 years of the Peruvian anchoveta *Engraulis ringens* [83]. The similarity on their life-span indicates that the cycles of collapse/recuperation of this *S aurita* stock might be similar to those of the Peruvian anchoveta.

Similar preference in distribution tied to upwelling plumes has been reported for other small pelagics. In California, *Sardinops sagax* is compressed along the coast during summer (non-upwelling season), while it is located offshore during upwelling [84]. During warm events the Namibian pilchard (*Sardinops sagax*) distribution is limited to small localized upwelling areas [57, 85]. In Perú, the pacific sardine (*Sardinops sagax*) is found farther offshore during winter/upwelling [86], but this distribution is compressed toward the coast during the primary stages of El Niño (warm) events [87]. All these species are exposed to a “perfect-storm” scenario of overfishing combined with prevalent ongoing environmental changes in the ocean.

Off northeastern Venezuela *Sardinella aurita* grow fast (K = 1.1 año^-1^) and reaches sexual maturity around 12–20 months of age [88, 89], so even a limited moratorium of this species can help the fisheries to recover. Fisheries management strategies need to evaluate the environmental conditions in order to provide regulations when changes in the environment affect the sardine habitat. For example, regulating fishery efforts when catchability increases due to natural factors, such as weak upwelling conditions, which also increase stressful conditions for this stock due to food scarcity. During the warm season, *S*. *aurita* biomass and number of schools tend to be higher closer to the coast, and therefore easier to be fished regardless of the total biomass of the stock. This information allows to prepare a management strategy when a weak upwelling is detected during the first trimester, regulating the stock fishery during the warm season on the second half of that year, in order to avoid further pressure on the stock or even overfishing.

## 5. Conclusions

Analyses of acoustic fisheries surveys conducted in the southeastern Caribbean Sea under the VECEP cooperative program showed that during upwelling conditions (December-April) the Spanish sardine, *Sardinella aurita*, was broadly distributed within upwelling plumes that extended from the coast across the continental shelf. In contrast, during the warm season (September-October) Spanish sardine was concentrated close to the coast within the first 10 km from the upwelling foci. During this time, sardines avoided warm waters, areas farther than 30 km from the upwelling foci, and the Orinoco River plume. Regardless of the upwelling season, higher numbers of schools were found in the cooler waters closer to the coast, especially during warm upwelling conditions.

The seasonal changes in the distribution of sardine provide insight into the causes of the collapse of the sardine fishery in the region in 2004, a fishery that still had not recovered by 2014. Sardine availability and catchability for the artisanal coastal fishery increases during warm conditions. The southern Caribbean Sea experienced substantial warming and decreased upwelling after 2004 [79]. One of the highest sardine catches of the last three decades also occurred in 2004, a year with anomalously weak upwelling and low phytoplankton concentrations. Subsequent years of weak upwelling added environmental pressures to the habitat of this short-lived stock due to food scarcity, while the strong fishery pressure limited the ability of the sardine to recover.

Sardine reproduces fast, so even a limited moratorium of this species can help the fisheries recovery. Fisheries management needs to implement the monitoring and evaluation of environmental conditions affecting the habitat of sardines in order to produce sound regulations of the stock. For example, during years with weak upwelling in the first trimester, particular regulations should be taken during the second half of the year to avoid further pressure and the potential collapse of the sardine stock.
